# Comprehensive Analysis of *BrDUF506* Genes Across the *Brassica rapa* Genome Uncovers Potential Functions in Sexual Reproduction and Abiotic Stress Tolerance

**DOI:** 10.3390/ijms252011087

**Published:** 2024-10-15

**Authors:** Guangqi Zhu, Jingxuan Wang, Shuang He, Kexin Liang, Renyi Zhang, Jiabao Huang, Xueqin Yang, Xiaojing Zhang

**Affiliations:** 1 College of Biology, Hunan University, Changsha 410082, China; 0923f17@zju.edu.cn; 2 Institute of Vegetable Science, Zhejiang University, Hangzhou 310058, China; 12416077@zju.edu.cn (S.H.); 22416232@zju.edu.cn (K.L.); renyizhang@zju.edu.cn (R.Z.); jiabaohuang@zju.edu.cn (J.H.); 3 College of Horticulture Science and Engineering, Shandong Agricultural University, Tai’an 271000, China; wangjingxuansdau@hotmail.com

**Keywords:** *Brassica rapa*, *DUF506*, bioinformatics analysis, abiotic stress, sexual reproduction

## Abstract

The Domain of Unknown Function 506 (*DUF506*) belongs to the PD-(D/E) XK nuclease superfamily and has been reported to play critical roles in growth and development as well as responses to abiotic stresses. However, the function of *DUF506* genes in *Brassica rapa* (*B. rapa*) remains unclear. In this study, a total of 18 *BrDUF506* genes were identified and randomly distributed across eight chromosomes, categorized into four subfamilies. Analyzing their promoter sequences has uncovered various stress-responsive elements, such as those for drought, methyl jasmonate (MeJA), and abscisic acid (ABA). *Bra000098* and *Bra017099* exhibit significantly enhanced expression in response to heat and drought stress. Protein interaction predictions indicate that Bra000098 homolog, At2g38820, is interacting with ERF012 and PUB48 and is involved in abiotic stress regulation. Furthermore, gene expression profiling has identified *Bra026262* with a high expression level in flowers and significantly decreased in female sterile mutants. Protein interaction prediction further revealed that its homolog, At4g32480, interacts with MYB and AGL proteins, suggesting the potential roles in female gametophyte development. The current study enhances our understanding of the functional roles of *BrDUF506s*, providing significant insights that are valuable in investigating sexual reproduction and abiotic stress responses in *B. rapa*.

## 1. Introduction

The Domain of Unknown Function (DUF) is a category of gene families that have conserved domains whose functions remain undetermined [1]. While many DUF families remain mysteries, research has been conducted on some. Proteins with DUF domains are involved in a range of biological processes, including modifying cell walls, producing lignin, and influencing microtubule dynamics [2,3,4]. This suggests a broad role for these proteins in various cellular functions.

Studies suggest that *DUF* genes are involved in various reactions to abiotic stress. The *OsDUF568* gene family, for instance, is essential for the growth of rice roots, leaves, and stems and is likely engaged in signaling pathways associated with abscisic acid and cytokinin, thereby enhancing the resistance of rice vegetative tissues to abiotic stress [5]. *OsMIZ1*, a member of the *OsDUF617* family, has been implicated in drought and salt stress tolerance, as well as in developmental and stress response processes in rice [6]. *ESK1*, a representative of the *AtDUF231* family, has been identified as a negative regulator in the context of freeze tolerance [7]. Furthermore, *DUF* genes also play a pivotal role in sexual reproduction. A study has identified causal genes for postzygotic reproductive isolation in rice hybrids, demonstrating that mutations in *DUF1668*-containing genes contribute to hybrid sterility and potentially drive speciation [8]. Another study has revealed that in seed plants such as *Arabidopsis* and tobacco, the HAP2/GCS1 fusogen interacts with sperm AtDUF679 membrane proteins DMP8 and DMP9, which are crucial for its translocation to the sperm plasma membrane in response to egg cell signals, thereby ensuring successful fertilization through a conserved mechanism [9].

The *DUF506* family is classified within the PD-(D/E)XK nuclease superfamily [10]. Research on the *DUF506* family has been conducted in *Arabidopsis* and *Oryza sativa* (*O. sativa*). AtRXR3, a phosphorus stress-inducible AtDUF506 protein in plants, negatively regulates root hair growth [11]. The *Arabidopsis AtDUF506* family is widely present in photosynthetic organisms and shows potential roles in coping with environmental stresses and nutrient shortages [12]. In rice, the *OsDUF560* family was actively engaged in the response to abscisic acid (ABA) and jasmonic acid (JA), exhibiting distinct expression profiles under drought and cold stress. This knowledge could be advantageous for future crop breeding efforts [13]. However, as of now, the *BrDUF506* genes in *B. rapa* have not undergone genome-wide identification, and their functions remain unexplored.

*B. rapa*, a vegetable extensively consumed within the Brassicaceae family, encounters dysgenesis and a range of abiotic stresses that undermine its productivity [14]. In light of the escalating global demand for vegetables, there is an imperative to identify genes capable of mitigating these issues and enhancing agricultural output. This study identified the *BrDUF506* genes at the genomic level and analyzed their physical and chemical properties, structure, gene, protein, and expression profiles. These findings offer critical insights for future research on *BrDUF506* genes in *B. rapa*.

## 2. Results

### 2.1. Identification and Physicochemical Characterization of DUF506 Gene Family in B. rapa

By comparing the homologous genes of the *AtDUF506* gene family with the *B. rapa* genome database, we found 18 *BrDUF506* genes and categorized them into four subfamilies. The genes were named based on their locations within the chromosomes. The physicochemical characteristics were summarized in Table 1. The *BrDUF506* genes are spread out over 8 of the 10 chromosomes. Bioinformatics analysis showed that the predicted isoelectric point (pI) ranged from 5.34 (Bra033896) to 10.78 (Bra040373), the protein lengths varied from 192 aa (Bra040373) to 367 aa (Bra033896), and MW ranged from 22 kDa (Bra040373) to 41 kDa (Bra033896). The length of the protein is linked to its molecular weight in a positive way. The majority of BrDUF506 proteins were predicted to be localized to the nucleus, with a few likelihoods in the cytosol and chloroplast (Table 1 and Supplementary Figure S1). Bioinformatics analysis suggests that BrDUF506 proteins might have various functions, depending on how they are distributed among the organelles.

### 2.2. Chromosomal Distribution and Phylogenetic Relationships of BrDUF506s

**Figure 1:**
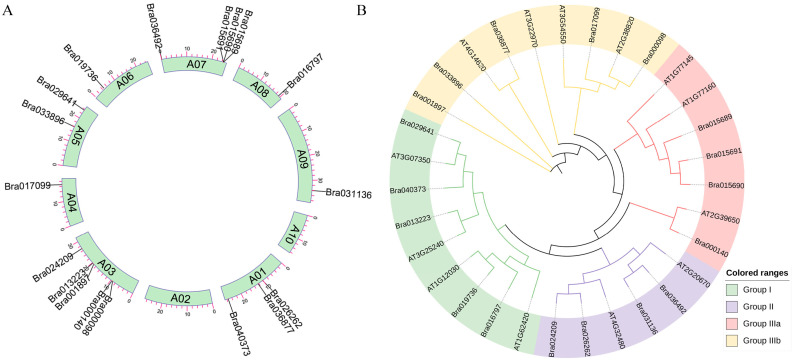
Chromosome localization and phylogenetic analysis of *BrDUF506* genes. (**A**) Chromosome localization of *BrDUF506s*, with chromosomes indicated in green bars, and black lines out of the colored box show *BrDUF506s* distribution in chromosomes. (**B**) Phylogenetic tree of *DUF506* gene family in *B. rapa* and *A. thaliana*. Green, purple, pink, and yellow correspond to Groups I, II, IIIa, and IIIb, respectively.

In total, eighteen *BrDUF506* genes have been identified in the *B. rapa* genome and are distributed non-uniformly across eight chromosomes, with chromosome 3 harboring the most *BrDUF506* genes, followed by chromosome 7, having four *BrDUF506* genes. While there are no *BrDUF506* genes distributed on chromosomes 2 and 9 (Figure 1A). In order to elucidate the evolutionary relationships among *BrDUF506* genes, we conducted a maximum likelihood (ML) phylogenetic tree of *DUF506* genes between *B. rapa* and *A. thaliana* (Figure 1B). The 31 *DUF506s* were clustered into four groups (I, II, IIIa, and IIIb); Group I and Group IIIb, comprising 9 members, were the largest groups. Group IIIa contained 7 *DUF506* members: 4 *BrDUF506s* and 3 *AtDUF506s*, and Group II was the smallest group, having 6 members (Figure 1B). Overall, the classification of *BrDUF506* members is largely concordant with the *A. thaliana* phylogenetic tree, suggesting a close relationship between *B. rapa* and *A. thaliana*.

### 2.3. Collinearity Analysis of BrDUF506 Members

**Figure 2:**
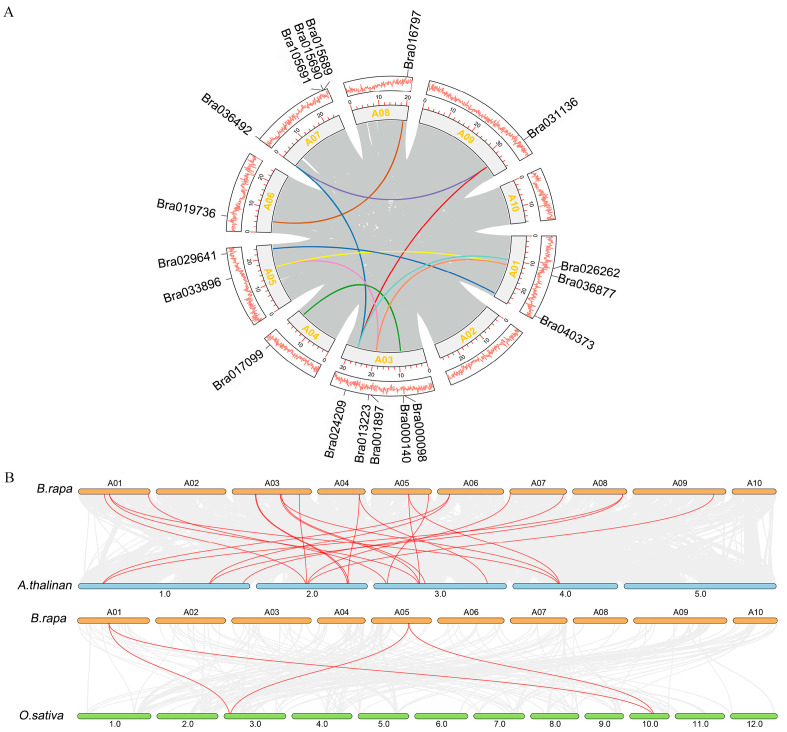
Synteny analysis of *DUF506s* in *B. rapa*, *A. thaliana*, and *O. sativa*. (**A**) Gray bars indicate the chromosomes of *B. rapa*. The orange lines in boxes represent the gene density of the chromosomes. Colored lines suggest duplicated gene pairs of *B. rapa*. (**B**) Chromosomal collinearity relationships between *B. rapa*, *A. thaliana*, and *O. sativa*. Orange, blue, and green indicate *B. rapa*, *A. thaliana*, and *O. sativa*, respectively. Red lines suggest collinear gene pairs.

Gene duplications are significant for gene family evolution [15]. The collinearity analysis result showed ten segmental duplications, with *Bra024209* harboring the most duplications: *Bra024209*-*Bra031136*, *Bra024029*-*Bra026262*, and *Bra024209*-*Bra036492*, followed by *Bra013223*, *Bra036492*, *Bra036877*, and *Bra033896* having two duplications, respectively (Figure 2A). This indicates that segmental duplication is a pivotal mechanism in the evolutionary expansion of the *BrDUF506* gene family.

In addition, to enhance our understanding of the origin and evolutionary history of *DUF506* genes, we also identified the duplicated events of *DUF506s* in *B. rapa*, the dicot model plant (*A. thaliana*), and the monocot species (*O. sativa*) (Figure 2B). Twenty-two pairs of collinear genes were identified between *BrDUF506* and *AtDUF506*, whereas only four pairs were found between *BrDUF506* and *OsDUF506* (Figure 2B). The findings reveal *BrDUF506s* have a greater degree of collinearity with *AtDUF506s* in comparison to *OsDUF506s*, indicating a significant relationship and functional similarity between *BrDUF506s* and *AtDUF506s*.

### 2.4. Characterization and Conserved Motifs of BrDUF506s

**Figure 3:**
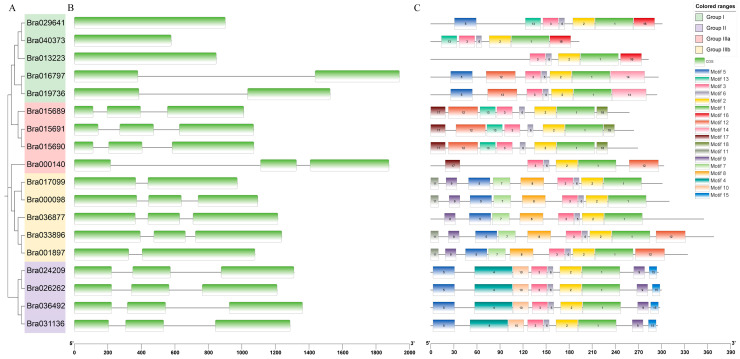
Gene structure and conserved motifs of *BrDUF506s*. (**A**) The phylogenetic tree of *DUF506* family in *B. rapa*; four different background colors indicate four subfamilies. (**B**) Exon-intron structure. Green rectangles and black lines indicate exons and introns, respectively. (**C**) Composition and distribution of conserved motifs in *BrDUF506s*. Motifs are shown by 18 different color bars.

A novel phylogenetic tree of BrDUF506 proteins was constructed through the analysis of full-length protein sequence alignments and categorized into four distinct groups (I, II, IIIa, and IIIb) (Figure 3A). It was noted that the BrDUF506 protein number exhibited a relatively even distribution across each of these groups. An analysis of the exon-intron configuration of the *BrDUF506* genes was performed, and it exhibits significant structural diversity, with *Bra029641*, *Bra040373*, and *Bra013223* in group I only having a single exon, while the other group I genes (*Bra016797* and *Bra019736*) harboring two exons (Figure 3B). Those *BrDUF506s* in groups II, IIIa, and IIIb possessed two or three CDS regions (Figure 3B). It suggested that members of different subfamilies might have distinct functions. Furthermore, eighteen conserved motifs of *BrDUF506s* were identified. These motifs vary in number and placement across *BrDUF506s*, but all *BrDUF506* members shared motifs 1, 3, 2, and 6 (Figure 3C). The conserved motifs in Group I exhibit the most significant differences, indicating potential functional diversification. Group II, IIIa, and IIIb each shared the same motifs, indicating the same subfamily may have the same biological function. In addition, motif 4 is only found in group II, and motif 7 is a group IIIb special (Figure 3C); this kind of structural similarity might hint at their specific functions in *B. rapa*.

### 2.5. Analysis of Cis-Acting Elements of BrDUF506s Promoter

**Figure 4:**
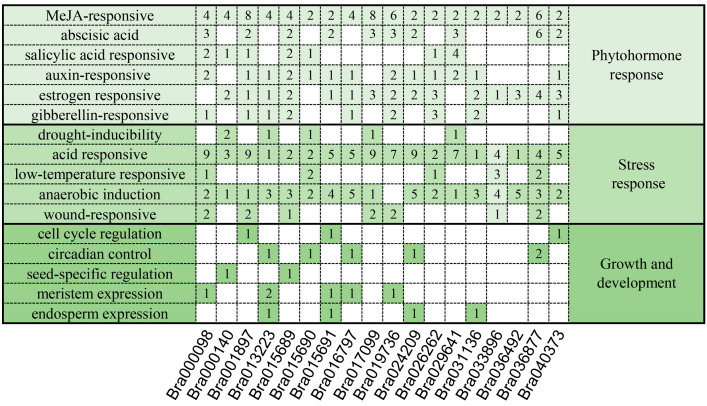
Distribution of *cis*-acting elements in *BrDUF506s* promoters.

Promoters act as essential “switches” for genes, triggering gene transcription and governing gene activity [16]. For further analysis of the prospective functions of *BrDUF506s*, we used PlantCARE to conduct an analysis of the 2 kb upstream region of the *BrDUF506s* coding region for *cis*-acting element prediction (Figure 4). Cis-acting elements were classified into three groups based on their function: growth and development, phytohormone response, and stress response. Figure 4 illustrates the quantity and arrangement of these elements for each gene. In the first category (phytohormone response), most genes had various phytohormone response elements, with 100% containing MeJA-responsive elements, 72.2% harboring auxin response elements, 55.5% having ABA-responsive elements, and the fewest containing GA-responsive elements, only 38.8% (Figure 4). Furthermore, the majority of *BrDUF506* genes possess estrogen-responsive elements, indicating their potential role in sexual reproduction (Figure 4).

In the stress response group, all *BrDUF506* genes contain acid response elements, and most genes harbor three or four types of stress response *cis*-acting elements, indicating that certain genes are key players in responding to stress. (Figure 4). The group IIIb gene *Bra000098* was found to encompass four different types of elements and boasts the highest number of elements. Similarly, other group IIIb genes *Bra017099*, *Bra036877*, *Bra033896*, and *Bra001897* each possess a variety of element types, with more than ten elements in total (Figure 4). Consequently, it is highly probable that the *BrDUF506* genes in Group IIIb play a significant role in tolerating abiotic stress.

### 2.6. Analysis of Abiotic Stress Transcript Levels of BrDUF506s

**Figure 5:**
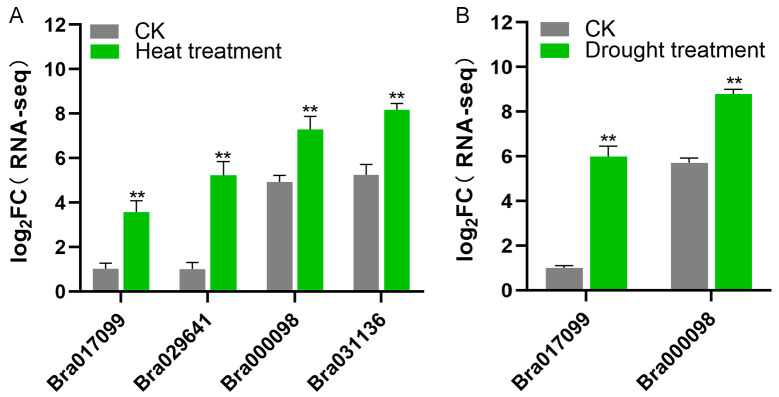
*BrDUF506s* are involved in abiotic stress tolerance. (**A**) Analysis of *BrDUF506* gene transcriptome data under heat treatment. Seedlings of *B. rapa* with six leaves were exposed to 28 °C lasting for 6 h before collection. (**B**) Analysis of *BrDUF506* gene transcriptome data under drought treatment. Seedlings of *B. rapa* with six leaves were subjected to a 15% PEG6000 solution to mimic drought conditions, with treatment lasting for 6 h before collection. Unstressed *B. rapa* seedlings with the same growth period and under identical growth conditions were used as a control (CK). All seedlings were harvested for RNA extraction and subsequent transcriptome analysis. Each group contained three biological replicates. Data are means ± SD; significant differences were determined by Student’s *t* test, ** *p* < 0.01.

To evaluate the role of the *BrDUF506* gene family in stress response mechanisms, we conducted an analysis of their genes transcriptome expression profiles subsequent to stress induction. The genes *Bra000098*, *Bra017099*, *Bra031136*, and *Bra029641* demonstrated significantly elevated expression levels in response to heat stress (Figure 5A; Supplementary Table S1). Furthermore, *Bra000098* and *Bra017099* also showed a notable increase in expression under drought stress conditions (Figure 5B). Additionally, a comparable trend is evident in *AtDUF506s* (Supplementary Figure S2). These results suggest that the *BrDUF506* gene family contributes to abiotic stress tolerance, with *Bra000098* and *Bra017099* being particularly noteworthy.

### 2.7. Expression Pattern of BrDUF506 Members in Different Tissues

**Figure 6:**
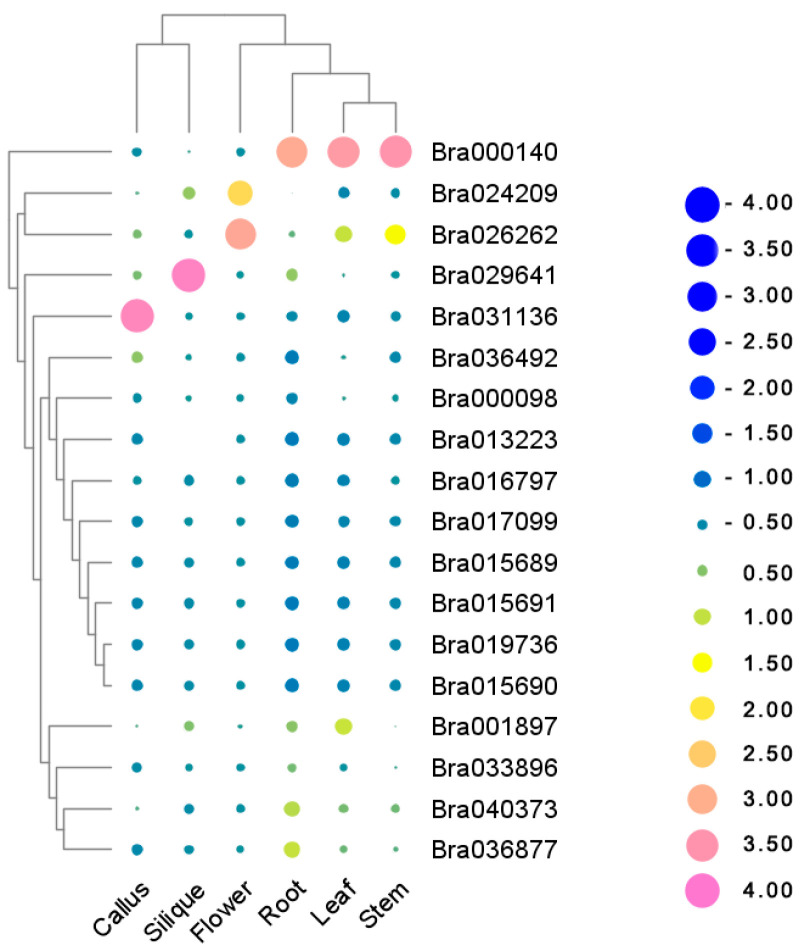
Tissue expression heatmap of *BrDUF506s*. All values underwent logarithmic transformation. Deeper pink, larger dots indicate higher expression level; deeper blue, larger dots indicate lower expression level.

An analysis of expression values in different tissues was performed to further investigate the expression specificities of the *BrDUF506s*. The data indicate that while the expression of these genes is generally low across most tissues, some genes see a significant increase in expression in specific tissues, suggesting they may have tissue-specific functions. Our analysis revealed that *Bra031136* was prominently expressed in callus, indicating that it potentially contributes to plant regeneration (Figure 6; Supplementary Table S2). Similarly, *Bra029641* displayed high expression in silique, but the homologous *Bra040373* does not have a similar expression pattern, presumably because they are evolutionarily different (Figure 6). Furthermore, *AT3G07350* was more related to *Bra029641* than *Bra040373*, thus *Bra029641* and *Bra040373* may have evolved different protein structures with diversity-conserved motifs that lead to different functions. Additionally, *Bra000140* was found to be highly expressed across various plant parts, including the root, leaf, and stem, possibly because its unique protein structure may be involved in a variety of biological functions (Figure 6). In flowers, both *Bra026262* and *Bra024209* showed high expression levels, suggesting group II genes are likely to be involved in sexual reproduction (Figure 6).

### 2.8. Sexual Reproduction-Related Expression Profiling of BrDUF506s

**Figure 7:**
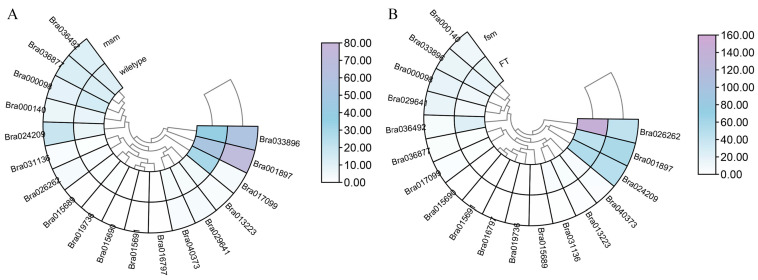
Expressions of *BrDUF506* in different sexual reproduction-related mutants. Analysis of the transcriptome data of *BrDUF506s* expression values in male sterile mutants (*msm*) (**A**) and female sterile mutants (*fsm*) (**B**) compared with wild-type (FT). The heatmap demonstrates the expression level; the color gradient from white to purple presents increasing expression values.

In order to determine whether *BrDUF506s* may be involved in sexual reproduction, we analyzed sexual reproduction-related expression profiling of *BrDUF506s* from the Chinese cabbage male sterile mutant (*msm*) and female sterile mutant (*fsm*) (Figure 7; Supplementary Table S3). *Bra001897* and *Bra033896* showed upregulation in *msm*, while *Bra017099* manifested an obvious down-regulated expression value in male sterile mutants (Figure 7A), indicating that these three genes may not only be involved in abiotic stress but also in sexual reproduction. Moreover, we found a distinct decline of *Bra026262* in *fsm* (Figure 7B), suggesting that it may regulate pistil growth and development.

### 2.9. Prediction of Protein-Protein Interaction (PPI) Network

**Figure 8:**
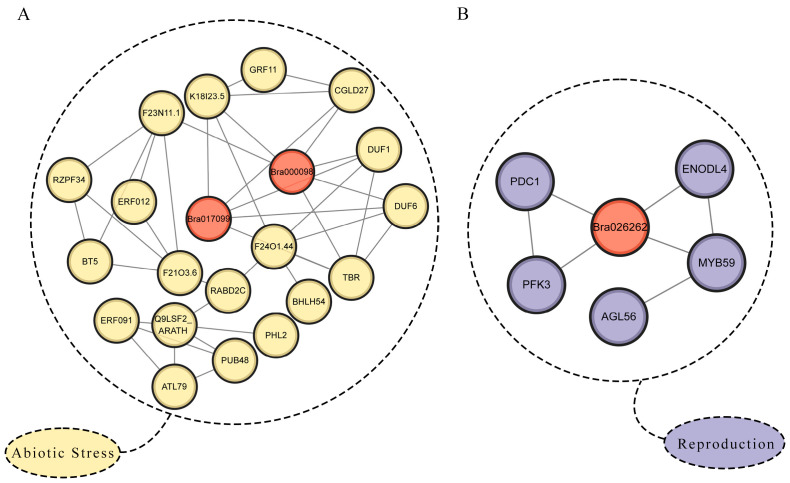
The PPI networks of DUF506 proteins in *B. rapa*. (**A**) The predicted Abiotic stress-related Bra017099 and Bra000098 PPIs. (**B**) The predicted sexual reproduction-related Bra026262 PPIs.

Proteins are fundamental in performing diverse cellular functions, and they physically interact with several types of molecules, including lipids, nucleic acids, and metabolites [17,18]. Considering the tight evolutionary bond between *B. rapa* and *A. thaliana*, we are making educated guesses about the biological roles of similar genes in *B. rapa* by analyzing how the *DUF506* genes in *A. thaliana* interact with proteins. The PPI network analysis utilized a public STRING database to conduct the predicted protein interaction map for three vital *BrDUF506* genes (*Bra017099*, *Bra000098,* and *Bra026262*) that are related to abiotic stress and sexual reproduction (Figure 8; Supplementary Table S4). We have identified some functional genes, including RZPF34 [19], ERF012 [20], and PUB48 [21,22] (Figure 8A), which are known to be involved in the ABA and ethylene response mechanisms. These genes are crucial in enhancing plant tolerance to abiotic stress. Interesting, we have also discovered genes *DUF1/6* (DUF724 domain-containing protein 1/6) (Figure 8A). Research on the DUF724 domain has shown its involvement in floral development [23]. We also identified genes MYB59 [24,25], PDC1 [26], and AGL56 [27] (Figure 8B), which are involved in plant growth and stress responses. This finding provides additional evidence of the functional diversity within the *BrDUF506* gene family.

## 3. Discussion

Consequent to anthropogenic activities and the phenomenon of climate change, there is an escalating incidence of multiple abiotic stresses on plant species. Such as severe temperature fluctuations, prolonged drought, excessive flooding, heightened light intensity, and modified salinity levels [28]. Furthermore, the issue of dysgenesis in plants is critically influencing the establishment and diversification of germplasm resources [29]. Previous research has underscored the vital importance of *DUF506s* in plant growth, development, reproduction, and coping with abiotic stress [11,12,13]. Nevertheless, the functional and family analysis of *DUF506* genes in *B. rapa* has not been previously investigated. In the present study, we utilized bioinformatics methodologies to examine the *BrDUF506* gene family features, gene expression profiles, and regulatory mechanisms. This research is anticipated to facilitate subsequent functional inquiries into the *BrDUF506* gene family.

This study identified 18 members of the *DUF506* gene family in *B. rapa*. Classification of these genes into four clusters was based on their phylogenetic relationships and sequence similarities. Collinearity analysis suggests that *BrDUF506s* is more closely related to *A. thaliana* than *O. sativa.*

Motif analysis has indicated the potential involvement of *Bra000098* and *Bra017099* in the regulation of responses to abiotic stress. Transcriptome data analysis has revealed a significant up-regulation of these genes in response to heat stress and drought conditions. Analysis of protein-protein interaction networks has revealed potential interactions among At2g38820 (homologs: *Bra000098* and *Bra017099*) and other cellular factors. Notably, an interacting protein, GRF11, is recognized as a critical regulatory component in the nitric oxide pathway, mediating responses to iron deficiency in *A. thaliana* [30]. At2g38820 interacts with CGLD27/At5g67370, which has been identified as important for the growth of *A. thaliana* under iron-limited conditions [31]. Studies have demonstrated that extracellular iron not only serves as an iron reservoir but also plays a significant role in modulating plant responses to environmental changes through targeted deposition [32]. Our research identified other interacting proteins, ERF012, an ethylene response factor, and PUB48, a member of the U-box protein family. ERF012 is involved in regulating stress responses through the modulation of auxin accumulation and ethylene biosynthesis [20]. Overexpression of *AtPUB48* has been shown to induce the expression of heat-related genes, including *HSP101*, *HSP70*, *HSP25.3*, *HSFA2*, and *ZAT12*, thereby enhancing plant resistance to heat stress during seed germination and seedling growth [22]. Moreover, *AtPUB46* and *AtPUB48* contribute to the regulation of plant sensitivity to drought and the inhibition of seed germination by abscisic acid (ABA), highlighting their significant roles in stress response mechanisms [21]. The above indicates that *BrDUF506s* may play a role in the regulation of abiotic stress responses through the mediation of hormones such as abscisic acid (ABA) and ethylene. Identify the potential pathways through which the *BrDUF506* gene family manages abiotic stress. Under conditions of heat or drought stress, Bra000098 and Bra017099 interact with ERF012 and PUB48, which in turn control genes that respond to stress, thus improving the plant’s ability to withstand environmental challenges.

*Bra026262* is potentially involved in the sexual reproduction of *B. rapa*, as indicated by its high expression levels in flowers and decreased expression in female sterile mutants. At4g32480 (homologs: *Bra026262* and *Bra024209*) may interact with MYB59, a member of the MYB family, and AGL56, a MADS box protein. *MYB* family members such as *MYB64/119* [33], *FLP* and *MYB88* [34], and *MYB98* [35] are known to regulate synergid cell differentiation, pollen tube guidance, and the formation of the filiform apparatus during female gametophyte development. MADS box proteins, including AGL51, AGL52, and AGL78, play roles in the female gametophyte [36]. Additionally, AGL9 and AGL15 recruit the FIS-PRC2 complex to regulate endosperm proliferation by modulating H3K27me3 marks and gene expression, suggesting a conserved role for MADS-box proteins in PRC2-mediated development across plants [37]. These findings suggest that Bra026262 may interact with MYB and AGL proteins, potentially recruiting the FIS-PRC2 complex to regulate female gametophyte growth and development.

## 4. Materials and Methods

### 4.1. Identification of DUF506 Members in B. rapa

The genome data file for *B. rapa* was retrieved from the BRAD database. (http://brassicadb.cn/), and the *A. thaliana* whole genome sequences were obtained from TAIR (https://www.Arabidopsis.org/), and the candidate *BrDUF506* members were identified through a two-way BLAST search of the *B. rapa* genome. Thirteen *Arabidopsis* DUF506 protein sequences were downloaded from the UniProtKB database (https://www.uniprot.org), and the eighteen BrDUF506 protein sequences were obtained from EnsemblPlants (http://plants.ensembl.org/), used as queries to perform protein blast searches by using TBtools [38]. Meantime, the typical domain of DUF506 (PDDEXK_6) was obtained from the Pfam database (http://pfam.xfam.org/) and was used to search for DUF506 with the HMMER tool (https://www.ebi.ac.uk/). The molecular weight (MW) and isoelectric point (pI) of BrDUF506 members were predicted with ExPASy (http://web.expasy.org/protparam/). Subcellular localization data were predicted with WoLF PSORT (Protein Subcellular Localization Prediction, https://wolfpsort.hgc.jp/).

### 4.2. Chromosomal Distribution, Phylogenetic Relationship, and Collinearity Analysis

The distribution of *BrDUF506* genes on the *B. rapa* chromosome was determined from *B. rapa* gff3 annotation files obtained from BRAD and plotted using TBtools software (v2.119). While the chromosome density was calculated using the Gene Density Profile plug-in in TBtools (v2.119) with default settings. The naming of *BrDUF506s* was based on their respective chromosomal locations.

Several Plug-ins, such as Advanced Circos, the Dual Synteny, and Table Row extract or filter in TBtools (v2.119) software, were used for synteny analysis. Collinearity relationships between duplicate genes within *B. rapa* and between *A. thaliana* and *O. sativa* were analyzed and visualized by TBtools.

Phylogenetic trees for the *DUF506* genes in *B. rapa* and *A. thaliana* were generated through maximum likelihood estimation utilizing the OmicShare platform. (https://www.omicshare.com/) by setting bootstrap to 1000, and further modified by using iTOL (https://itol.embl.de/) tools.

### 4.3. Conserved Motif, Gene Structure, and Cis-Element Prediction of BrDUF506 Family Genes

The conserved motifs were analyzed and predicted with MEME (http://meme-suite.org/) and visualized by TBtools (v2.119). Visualization of the gene structure of *BrDUF506s* was achieved through the application of the TBtools basic Visual Gene Structure program.

The *BrDUF506s*’ promoters were obtained through EnsemblPlants Family gene initiation codon 2000 bp upstream sequences and used to search for *cis*-elements with the PlantCARE database (http://bioinformatics.psb.ugent.be/webtools/plantcare/html/) with default parameters.

### 4.4. Gene Expression Analysis

*BrDUF506s* tissue-specific expression data in different tissues (callus, flower, leaf, root, silique, and stem tissues) were extracted from BRAD (http://brassicadb.cn/) and visualized using TBtools (v2.119). *AtDUF506s* expression data under various abiotic stress treatments were downloaded from the *Arabidopsis* eFP Browser (http://bar.utoronto.ca/). For significant data, heatmap matrices were plotted using TBtools.

The transcriptome data of *B. rapa* in two important mutants associated with sexual reproduction (male sterility mutant, msm, and female sterility mutant, fsm) was obtained from NCBI GEO (https://www.ncbi.nlm.nih.gov/geo/) with accession numbers GSE125485 and GSE147438 and normalized using the transcripts per million (TPM) method. Gene expression heatmaps were plotted using TBtools.

### 4.5. Plant Growth Conditions, Abiotic Stress Treatments, and Transcriptome

*B. rapa*-cultivated species with stable self-incompatibility was used for expression analysis. The plump seeds were sown in MS medium and cultivated in a plant incubator. Six leafy seedlings were selected for abiotic stress treatments. The seedlings were grown in a hydroponic system with 150 mM NaCl to simulate salt stress and in 15% PEG6000 for drought conditions. The seedlings were exposed to 4 °C for cold stress and 28 °C to simulate heat stress. At the same time, used similar growth status untreated *B. rapa* seedlings as a control (CK). All materials were collected for RNA extraction and transcriptome analysis. A minimum of three biological replicates were performed for each treatment.

### 4.6. Prediction of Protein-Protein Interaction Networks

Predictions for the protein-protein interaction network analysis were performed utilizing the STRING website (https://cn.string-db.org/) with default parameters, and Cytoscape v3.10.2 was used to construct the interaction network.

## 5. Conclusions

**Figure 9:**
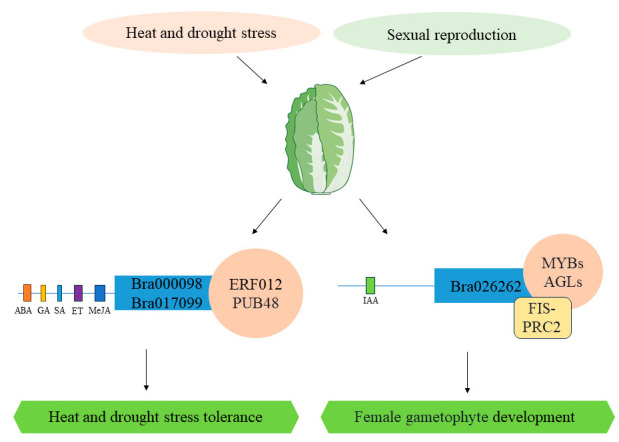
The putative molecular mechanism underlying the regulation of stress tolerance and sexual reproduction by *BrDUF506s*.

In conclusion, the comprehensive analysis of genome-wide identification, expression profiling, and protein-protein interactions suggests that *Bra000098* and *Bra017099* are potential regulators of heat and drought stress tolerance, while *Bra026262* is involved in the regulation of female gametophyte growth and development (Figure 9). These findings provide valuable insights into the functional roles of these genes in *B. rapa*, which is essential for devising strategies to increase crop resilience and inform breeding practices. Through the application of gene knockout or overexpression techniques, we can precisely gauge the impact of these genes on stress resistance and reproductive functions, underscoring their potential in crop improvement. These genes could be promising targets for marker-assisted selection in breeding programs aimed at enhancing crop resilience. The targeted identification and selection of gene variants that correlate with enhanced stress tolerance can enable breeders to cultivate crop varieties with improved capabilities to endure rigorous environmental stressors. In summary, the results presented in this study have the potential to significantly advance our understanding of gene function in Brassica rapa and to drive innovation in crop breeding, ultimately contributing to the development of more resilient and high-yielding varieties.

## Figures and Tables

**Table 1:** Characteristics of the *BrDUF506* gene family.

Gene ID	Chromosome (Chr)	Start	End	pI	Molecular Weight (Average)	Subcellular Location	Protein Length (aa)	*A. thaliana* *DUF506* Genes
Bra026262	A01	10,328,544	10,330,239	6.96	33,655.44	nucl	299	AT4G32480
Bra036877	A01	12,460,526	12,461,742	5.69	39,571.58	cyto	354	AT4G14620
Bra040373	A01	27,947,913	27,948,491	10.78	21,600.19	chlo	192	AT3G07350
Bra000098	A03	9,236,560	9,237,656	8.38	34,659.74	nucl	309	AT2G38820
Bra000140	A03	9,501,674	9,503,552	7.04	33,728.35	nucl	302	AT2G39650
Bra001897	A03	19,213,110	19,214,189	5.55	36,930.09	cyto	333	AT3G22970
Bra013223	A03	19,776,413	19,777,261	6.55	31,935.97	nucl	282	AT3G25240
Bra024209	A03	26,866,824	26,868,136	6.99	33,163.73	nucl	295	AT4G32480
Bra017099	A04	16,615,630	16,616,603	8.7	33,762.84	nucl	300	AT2G38820
Bra033896	A05	14,926,740	14,927,979	5.34	40,567.86	cyto	367	AT3G22970
Bra029641	A05	22,850,133	22,851,035	6.54	33,558.79	nucl	300	AT3G07350
Bra019736	A06	4,719,393	4,720,920	6.21	33,768.01	cyto	293	AT1G12030
Bra036492	A07	88,234	89,597	6.9	33,380.91	nucl	297	AT2G20670
Bra015691	A07	21,336,474	21,337,545	8.76	30,140.59	nucl	263	AT1G77145
Bra015690	A07	21,338,681	21,339,754	8.69	30,837.18	nucl	268	AT1G77145
Bra015689	A07	21,345,306	21,346,318	8.76	29,586.01	nucl	257	AT1G77145
Bra016797	A08	20,033,880	20,035,821	7.56	33,604.24	cyto	295	AT1G12030
Bra031136	A09	32,332,835	32,334,124	7.53	33,184.72	nucl, cyto_nucl	294	AT2G20670

aa, amino acids. Subcellular location: chlo (chloroplast), cyto (cytosol), nucl (nucleus).

## Data Availability

All the data that support the findings of this study are available in this paper and its Supplementary Material published online.

## Supplementary Materials

The following supporting information can be downloaded at: https://www.mdpi.com/article/10.3390/ijms252011087/s1.
