# Supplementary material for: Comprehensive Analysis of BrDUF506 Genes Across the Brassica rapa Genome Uncovers Potential Functions in Sexual Reproduction and Abiotic Stress Tolerance
Source: Int J Mol Sci. 2024 Oct 15;25(20):11087. doi: 10.3390/ijms252011087 (PMC11507830; doi:10.3390/ijms252011087)
Supplement: Supplementary file 1 [file ijms-25-11087-s001.zip › Supplementary Materials .pdf]

Supplementary Material. Zhu et al. (2024). The comprehensive analysis of *BrDUF506s* across the *Brassica rapa* genome underlying the potential functions in enduring abiotic stress and facilitating sexual reproduction

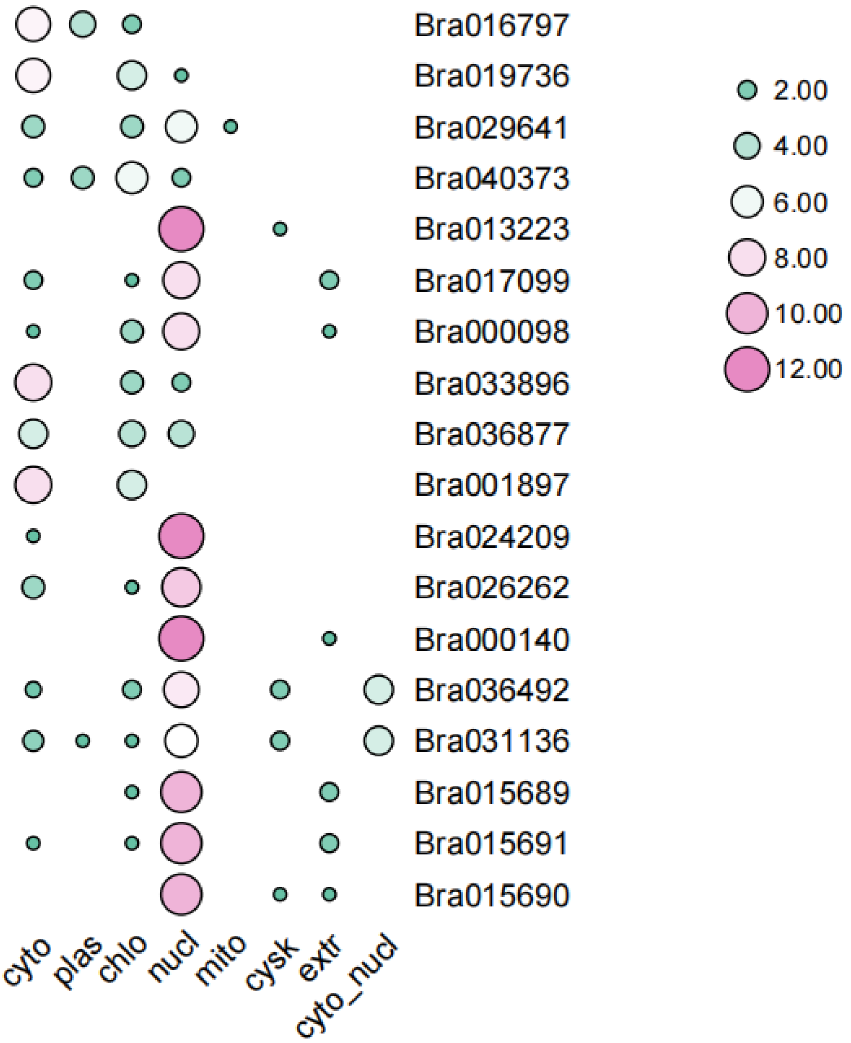

Figure S1. Predictive analysis of BrDUF506 proteins subcellular localization: cyto (cytosol), plas (plasma membrane), chlo (chloroplast), nucl (nucleus), mito（mitochondria）,cysk (cytoskeleton), extr (extracellular),.Larger area and darker color indicate higher express.

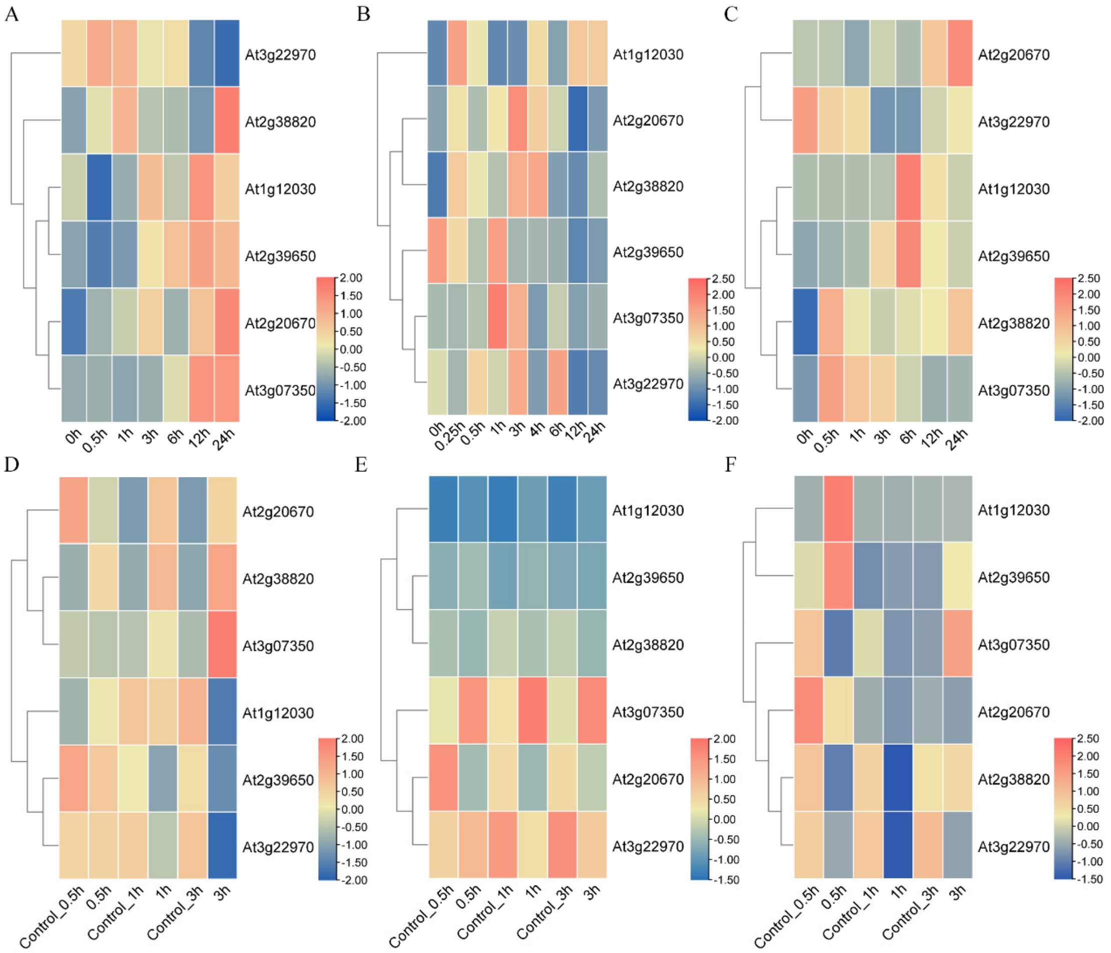

Figure S2. Expression analysis of *BrDUF506s* homolog in *A. thaliana* upon environmental unfavorable conditions. The expression pattern of the *AtDUF506s* was investigated under Cold (4℃) (A), Heat (28℃) (B), Salt (150 mM NaCl) (C), ABA (D), MeJA (E), IAA (F). All values underwent logarithmic transformation. Color scale represents fold changes, where red color indicated upregulation and blue color indicated downregulation of individual gene.
